# Cuticular Wax Triterpenes Maintain Storage Quality of Blueberries by Reducing Water Loss

**DOI:** 10.3390/foods12142643

**Published:** 2023-07-08

**Authors:** Qi Kong, Ruiling Liu, Weijie Wu, Xiangjun Fang, Hangjun Chen, Yanchao Han, Jianye Chen

**Affiliations:** 1 State Key Laboratory for Conservation and Utilization of Subtropical Agro-Bioresources/Guangdong, Provincial Key Laboratory of Postharvest Science of Fruits and Vegetables/Engineering Research Center of Southern Horticultural Products Preservation, Ministry of Education, College of Horticulture, South China Agricultural University, Guangzhou 510642, China; 18363900697@163.com; 2 Key Laboratory of Post-Harvest Handing of Fruits, Ministry of Agriculture and Rural Affairs, Key Laboratory of Fruits and Vegetables Postharvest and Processing Technology Research of Zhejiang Province, Key Laboratory of Postharvest Preservation and Processing of Fruits and Vegetables, China National Light Industry, Food Science Institute, Zhejiang Academy of Agricultural Sciences, Hangzhou 310021, China; ruilingliu2005@163.com (R.L.); wuweijie87@163.com (W.W.); fangxiangjun2004@163.com (X.F.); hang_junchen@163.com (H.C.)

**Keywords:** blueberry, cuticular wax, triterpenoids, storage quality, hydrophobicity, moisture migration

## Abstract

Cuticular wax contributes to maintaining postharvest storage quality against fruit water loss and softening. Triterpenoids, such as oleanolic acid (OA) and ursolic acid (UA), are the main components in blueberry cuticular wax, but their role in water migration during the storage of blueberries remains to be determined. Here, we examined the relationship between the content of OA and UA and the storage quality of blueberry fruit (25 °C). The results revealed that the UA content during eight-day postharvest storage ranged from 58 to 77 $\mu g\;{cm}^{-2}$, which was negatively related to weight loss. Additionally, we investigated the effect of exogenous OA and UA on water migration in the blueberry fruit during storage at room temperature; the weight loss was significantly lower (by 22%) with UA treatment than in the control fruit. Our findings indicate that OA and UA effectively affect water migration in blueberry fruit during postharvest storage, which could contribute to improving postharvest preservation techniques.

## 1. Introduction

As the outer barrier between the environment and higher plants, the structure, morphology and function of the cuticle have been studied extensively [1]. Esterified fatty acids and cuticular wax make it highly hydrophobic. Cuticular wax is a crucial component of the cuticle and serves as the primary physical barrier dedicated to resisting varying external environmental stresses, such as regulating non-stomatal water loss, reflecting UV-radiation, avoiding extreme temperature damage, and providing a self-cleaning mechanism, as well as preventing pathogens and insect infection [2]. Therefore, a mounting number of researchers have pointed out that cuticular wax is strongly associated with the postharvest quality performance of fruit [3,4].

Blueberries have a powdery wax that coats their bluish-black skin, which can effectively prevent fruit from cracking and maintain texture, sensory, and nutritional quality [5]. Chu et al. [4] found that removing the cuticular wax on blueberries accelerated water loss and softening as well as a decline in apparent and physiological qualities. In addition, it is worth noting that the main defects of blueberries are usually fruit softening and dehydration during storage, seriously affecting consumer acceptance and their market value [6]. Some studies on blueberry fruit softening have concentrated on cell wall metabolism, and other potentially related factors’ specificity remain unclear. Interestingly, a handful of recent research has shown that water loss was one of the factors affecting firmness changes in postharvest fruit [7,8], and noticeable differences vary among different species. It is well known that cuticular wax is one of the determinants in water-proofing barrier performance [9,10]. According to several previous reports, the removal of citrus fruit cuticular wax significantly promoted postharvest moisture loss [11], while maintaining a high cuticular wax amount in pepper fruit during storage contributed to the reduction of moisture loss [12]. Additionally, triterpenoids and sterols contained in cuticular wax also significantly influenced the postharvest moisture loss performance of pepper fruit [13]. However, cuticular wax was a kind of complex mixture, the specific components involved in the moisture migration process of blueberry fruit are largely unknown.

Cuticular wax is a complex mixture mainly made up of very-long-chain aliphatics, including fatty acids, alkanes, aldehydes, ketones, and alcohols, as well as alicyclic compounds such as triterpenoids and aromatics [14]. These compositions allow great variations among and within different species and cultivars in response to varying external environments [5,15]. Triterpenoids are the most abundant components in blueberry cuticular wax, and ursolic acid (UA) or oleanolic acid (OA) are authenticated as the major triterpenoids among nine commercial cultivars [16]. These have been shown to be highly hydrophobic and can improve the physical stability of the cuticular wax water barrier. Recently, studies on tomatoes, sweet cherries, and peaches have indicated a significant correlation between water loss performance and the proportion of n-alkanes to triterpenoids [17,18,19]. Moggia et al. [6] also demonstrated that triterpenoids (UA contents) in the cuticular wax of the highbush blueberry have a potential effect on fruit weight loss and softening performance. Interestingly, the blueberry cuticular wax content (especially the content of triterpenoids) continuously increased under low temperature stress (at 0.5 °C and 95% RH) [20], which contrasted with the blueberry storage at 4 °C and 90% RH [21]. However, how the changes in cuticular wax components UA and OA influence water transport and migration during storage of blueberries at room temperature (25 °C) needs to be elucidated.

Therefore, the work reported herein is intended to investigate the changes in cuticular wax components UA and OA in blueberry fruit during postharvest storage (at 25 °C and 85% RH) and then, to reveal the fruit water migration process after being treated by exogenous UA and OA. These results will help to explain the functional role of cuticular wax components in the postharvest preservation of fruit.

## 2. Materials and Methods

### 2.1. Fruit Sampling

Blueberry fruit (*Vaccinium ashei* cv. “Britewell”) was harvested from a commercial field located in Anji, China. Samples for wax extraction were carefully collected with scissors and placed directly into a conical flask; each bottle contained ten fruit (a total of 15 bottles were used for subsequent wax extraction). Other samples were placed in plastic clamshells and sealed in a biological ice pack foam box and transported to the laboratory via refrigerated truck. Samples without visible wounds or disease, as well as of uniform size were picked for follow-up experiments. All samples were randomly selected and divided into 125 g plastic clamshells (containing 30 fruit each), stored at 25 °C, 85% relative humidity (RH) for eight days. Then, they were sampled every two days for subsequent index determination and cuticular wax extraction.

In order to conduct OA and UA treatment experiments, fifteen boxes were randomly divided into three groups (CK, OA, and UA treatment groups), three boxes were used only for weight loss measurement, and the other two boxes were randomly sampled (six fruit each) for water migration analysis. The OA and UA (Shanghai Macklin Biochemical technology Co., Ltd., Shanghai, China) treatment method was according to Yang et al. [22] and slightly modified: samples were treated by coating with a morpholine fatty acid salt. The emulsion system (containing morpholine fatty acids, OA, and UA) was prepared by using Tween 80 (Shanghai Aladdin Biochemical technology Co., Ltd., Shanghai, China) as surfactant under the condition of continuous heating and agitation. The sample coated with morpholine fatty acid salt alone was Control (CK), and the morpholine fatty acid salt supplemented with 10% OA and 10% UA (*w*/*w*), respectively, was treated as the experimental group. Two coats were applied evenly to each fruit and stored at 25 °C, 85% RH. The weight-loss rate and moisture migration of samples were measured every two days.

### 2.2. Wax Extraction and Determination

A total of 30 mature samples were taken for total wax extraction based on the method performed by Chu et al. [16]. An extract of ten blueberries was obtained by dipping them twice in 10 mL chloroform for 1 min at room temperature, then filtered by quantitative filter paper and dried under nitrogen flow at 40 °C. The dried extract is the total wax (μg). The experiment was conducted as fully randomized and triplicated.

Fruit surface area after wax extraction was determined according to the method of Ketata et al. [23]. Briefly, the equatorial diameter [*d*_1_] and polar diameter [*d*_2_] of the fruit were determined by digital vernier caliper. The surface area (${cm}^{2}$) =$\;4\pi r^{2}$, $r=\left(d_{1}+d_{2}\right)/4$. Wax content was recorded as microgramme per unit ($\mu g\;{cm}^{-2}$).

High-performance liquid chromatography (HPLC) analysis was performed on Agilent 1200 series, Eclipse XDB-C18 column (250 × 4.6 mm, 5 μm). The dried extract was made up to exactly 5 mL with methanol and filtered through a 0.22 μm membrane filter. 

A mixed standard solution was prepared by OA and UA standards with 5 mL methanol. The standard working solutions contained 0.05, 0.1, 0.15, 0.2, 0.25, and 0.3 $mg\;{mL}^{-1}$ of OA and 0.1, 0.2, 0.3, 0.4, 0.5, and 0.6 $mg\;{mL}^{-1}$ of UA. The mobile phase of methanol: water: acetic acid: triethylamine = 265:35:0.1:0.05 (*v*/*v*/*v*/*v*) was used for sample testing; some test conditions were followed: flow rate (1.0$\;mL\;{min}^{-1}$), detection wavelength (210 nm), column temperature (35 °C), and sample quantity (20 μL). 

### 2.3. Quality Assessments

Fruit firmness was measured based on the method performed by Jiang’s method [24]. Ten samples were chosen randomly and compressed to pedicle area of 5 mm under speed of 1.0 mm s^−1^. The peak force (N) during the test represents fruit firmness. A total of 90 samples (three boxes) were selected to determine the water-loss rate. The samples were weighed as *W*_1_ and *W*_2_ before and after storage, and the results were then calculated by the following formula: (%) $=\left[\left(w_{1}-w_{2}\right)/w_{1}\right]\times 100$ [25]. The diameter of the sample was measured in the same way as the surface area.

The scanning electron microscope (TM 3000, Hitachi, Tokyo, Japan) was used to observe the cuticular wax shape of blueberry fruit. Blueberry pericarp pieces (3 mm × 3 mm) were excised from the equatorial surface of blueberries with a blade and dried in a vacuum freeze dryer. The dried sample was then sputter coated with gold (20 nm thick) in an ion sputter coater (SBC-12, KYKY, KYKY Technology Co., Ltd., Beijing, China) [16].

### 2.4. Hydrophobic Properties

The water contact angles of OA and UA were quantified using Data Physics OCA-15EC [26]. The powdered samples of OA and UA were made into a round slice (radius was 1 cm) before contact angle measurements. A volume of 3 µL of deionized water droplet was placed on the circular sample sheet. The angle at which the droplet touched the sample was then recorded. The results were derived from three independent experiments.

The polarity of the two components was reflected by the diffusive position of the thin chromatography silica gel plate. The starting and ending lines were marked 1 cm from each end of the silica gel plate. The solvent system used for developing the TLC plates is petroleum ether: ethyl acetate = 2:1 (*v*/*v*). The samples were spotted on the starting line with a capillary pipette. After the samples were expanded by unfolding agent, the color was developed using a 95% sulfuric acid ethanol solution.

### 2.5. Measurement of Moisture Migration

According to the method performed by Zhu et al. [27], low-field nuclear magnetic resonance (LF-NMR) relaxation measurements of the samples were conducted on a MacroMR (MacroMR12-150V-I, Suzhou Niumag Analytical Instrument Corporation, Suzhou, China) with a neutron resonance frequency of 12 MHz. The standard water samples were placed into the magnet coils for individual samples, and the instrument automatically adjusted the center and RF frequency. Then, the blueberry samples were placed in the center of the magnetic field, and the transverse relaxation time (T_2_) was determined by the Carr–Purcell–Meiboom–Gill (CPMG) sequence with *τ* value of 300 μs. Data from 12,000 echoes were acquired at 32 °C. The repetition delay between two subsequent scans was 3000 ms.

### 2.6. Data Analysis

Statistical analyses of the data were executed with the SPSS software V23.0. The significance was assessed using Duncan’s multiple range test (*p* < 0.05), then mapped using Origin 2017. The experiment was conducted as fully randomized and triplicated, and the data were presented as means ± SD.

## 3. Results

### 3.1. Determination of Total Wax Content and Storage Quality Performance

**Figure 1:**
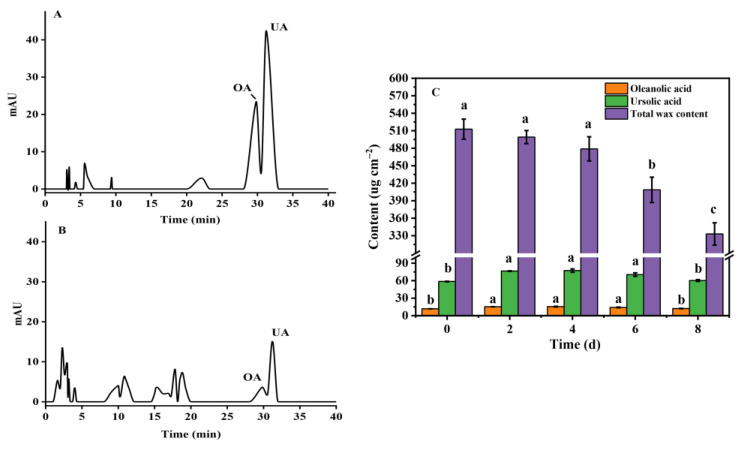
Changes in total wax and triterpenoid composition content during storage at room temperature (25 °C) in blueberry. (**A**) and (**B**) were HPLC peak spectra of the standard and sample, respectively, suggesting that the mobile phase used in the experiment can separate the two components. (**C**) Changes in total wax and triterpenoid composition content. Data are presented as means ± SD. Letters show significance (*p* < 0.05).

**Figure 2:**
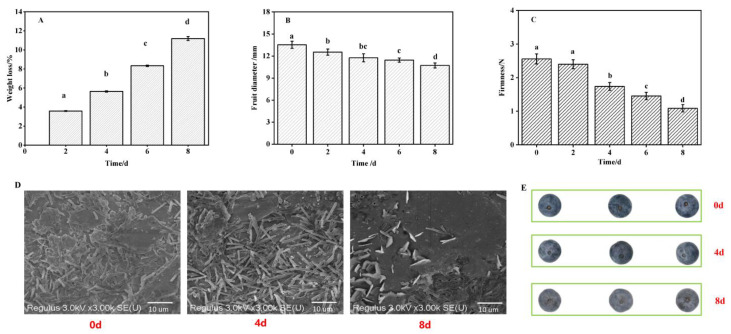
Storage quality performance of fruit: (**A**) weight loss, (**B**) fruit diameter, (**C**) firmness, (**D**) status of cuticular wax, and (**E**) appearance of samples. The image of the status of cuticle wax in blueberry fruits has been magnified 3000 times. In (**A**–**C**), mean differences between the bars are significant at *p* < 0.05 level for different letters but not significant for the same letters.

Chloroform extracts were used to obtain total wax as well as two triterpenoid acids (OA and UA). Figure 1A,B showed that the mobile phase of HPLC can accurately separate OA and UA, and the content changes in both were shown in Figure 1C. As can be seen from Figure 1C, the total amount of wax continued to decrease with the passage of storage time, while there was no significant difference in the first four days of storage. The amount of wax present during storage decreased constantly between 512 and 333 $\mu g\;{cm}^{-2}$; by the end of storage, the wax amount had been reduced to 65% of its initial quantity. To study the effects of cuticular wax and component changes on the storage quality of blueberries, especially on the water loss and softening, we measured the weight loss, hardness, fruit diameter, and wax appearance of blueberry fruit during storage (Figure 2). Obviously, it was expected that the weight-loss rate increased significantly and reached 11% at the end of storage (Figure 2A). The fruit diameter (Figure 2B) and firmness (Figure 2C) decreased to 79% and 48% of their initial storage levels, respectively, along with smoothing out the waxy epidermis (Figure 2D). According to these results, wax content in the fruit epidermis may have a significant impact on the exterior quality during storage. Therefore, we hypothesized that the wax content may have an effect on the process of water migration.

### 3.2. Correlation between Storage Qualities and Contents of Triterpenoid Substance OA and UA

**Figure 3:**
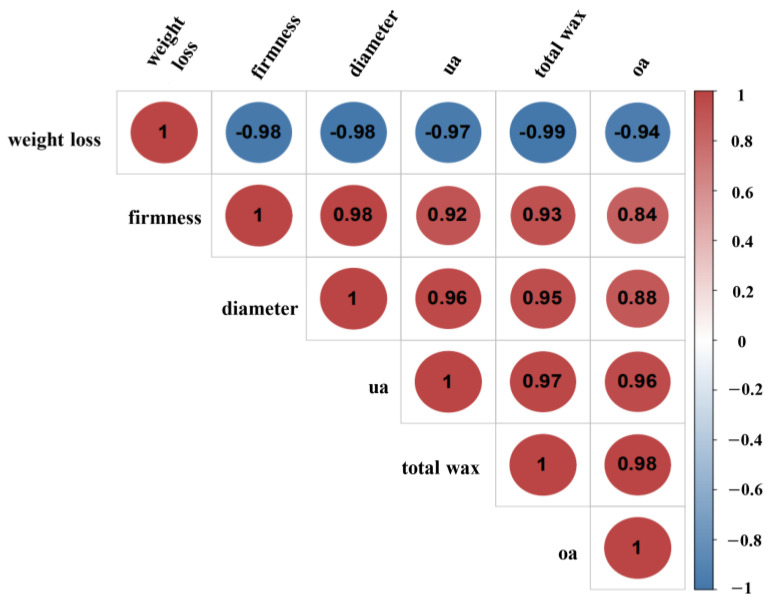
Correlation coefficients between fruit characteristics and wax content. Pearson correlation coefficient calculation method was used to establish correlation (*p* < 0.05). The red zone demonstrates a positive correlation between two variables, the blue suggests a negative correlation between two variables, and the numbers in each cell indicate the correlation coefficient.

Triterpenoids are major components in blueberry cuticular wax, mainly OA and UA [16]. According to Figure 1A,B, the peak spectra of HPLC was used to determine the contents of OA and UA. As Figure 1C showed, UA content was higher than OA, and it appears that the changes in OA and UA were not fully consistent with the changes in total wax content, as the content of UA and OA increased during the first four days of storage when the total wax was unchanged. These different evolutions could be attributed to different metabolic processes among complex components of cuticular wax. Interestingly, the content at the end of storage was not significantly different from that at the beginning. A typical UA sample had an initial storage content of about 58 $\mu g\;{cm}^{-2}$, increased to about 77 $\mu g\;{cm}^{-2}$ after four days of storage, and decreased to 60 $\mu g\;{cm}^{-2}$ after the last day of storage, which amounted to no significant changes compared with the beginning of storage. After four days of storage, the total amount of wax gradually decreased; UA and OA showed the same trend, with a significant decrease on the sixth day. Subsequently, we conducted correlation analysis between fruit storage quality indices (weight loss, hardness, fruit diameter) and changes in total wax and OA and UA contents (Figure 3). The results showed that there was a significant correlation (*p* ≤ 0.05) between fruit quality and wax change during storage. A negative relationship was found between persistent water loss during storage and wax amount, UA contents, fruit diameters, and hardness (r < −0.95). Moreover, as the most abundant components of blueberry wax (OA and UA), the changes in UA and OA content were closely related to the wax content, despite some differences in wax content and their changing trends during storage. These results indicated that the contents of total wax and UA may be closely related to the storage quality of blueberries, especially affecting the water loss, shrinkage, and softening of blueberries during storage.

### 3.3. Hydrophobic Characteristics of OA and UA 

**Figure 4:**
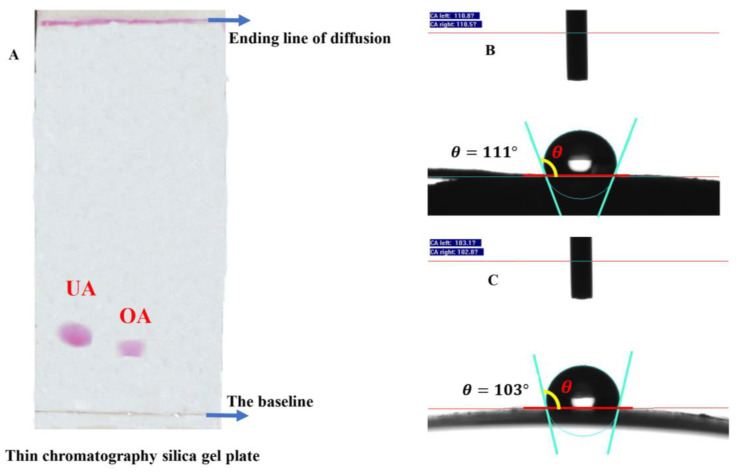
Hydrophobic characterization of OA and UA. (**A**)The polarity of the two components was reflected by the diffusion position of the thin chromatography silica gel plate, the faster the rate of diffusion, the less polar the component. (**B**) and (**C**) were the droplet contact angles of UA and OA, respectively. Water droplet contact angle greater than 90 is hydrophobic material; the larger the angle, the stronger the performance.

To explore the hydrophobic characteristics of the wax components OA and UA, we characterized the two components by molecular polarity experiments and water droplet contact angles. As can be seen from Figure 4A, for OA and UA in the same polarity diffusion agent, thin-layer chromatography on silica gel plate diffusion rate and position showed that UA has a lower molecular polarity, indicating that UA has stronger hydrophobic ability; when attached to the blueberry fruit skin, it may be more likely to form a dense hydrophobic protective layer, helping to suppress the water loss of the fruit. Meanwhile, the analysis after measuring the droplet contact angles of the two components also showed that although both of them were larger than 90 and had hydrophobic ability, the droplet contact angle of UA was larger than that of OA, suggesting that UA has a stronger hydrophobic ability (Figure 4B,C). Based on these results, UA was more hydrophobic with lower polarity than OA, despite their both being hydrophobic. Therefore, we hypothesized that blueberry fruit may benefit from the treatment of the wax components (OA and UA) during storage by slowing down water migration and retaining water in storage, extending its shelf life.

### 3.4. Effects of OA and UA on Fruit Moisture Migration

**Figure 5:**
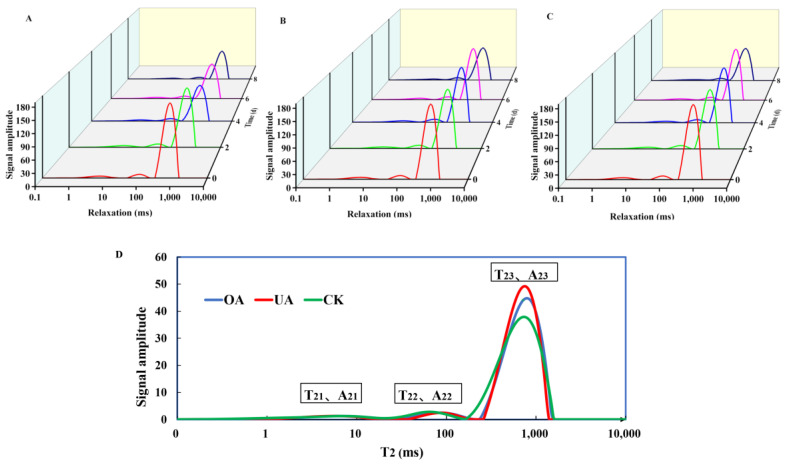
LF-NMR T_2_ relaxation time curve of blueberries during storage. (**A**–**C**) were the changes in signal amplitudes of different water molecules in blueberry fruits treated with CK, OA, and UA, respectively. The signal amplitude was positively correlated with the content. (**D**): T_21_, T_22_, and T_23_ refers to the transverse relaxation times of the bound water and free water molecules, respectively. A_2i_ is the peak area of T_2_ signal inversion of different component, which can reflect the component content. It can be seen that the peak area of T_23_ of all samples is the largest, indicating that the dominance of the free water proportion. In (**A**), (**B**) and (**C**), the five colors represent the peak spectra at 0, 2, 4, 6 and 8 days, respectively, corresponding to the time axis.

**Figure 6:**
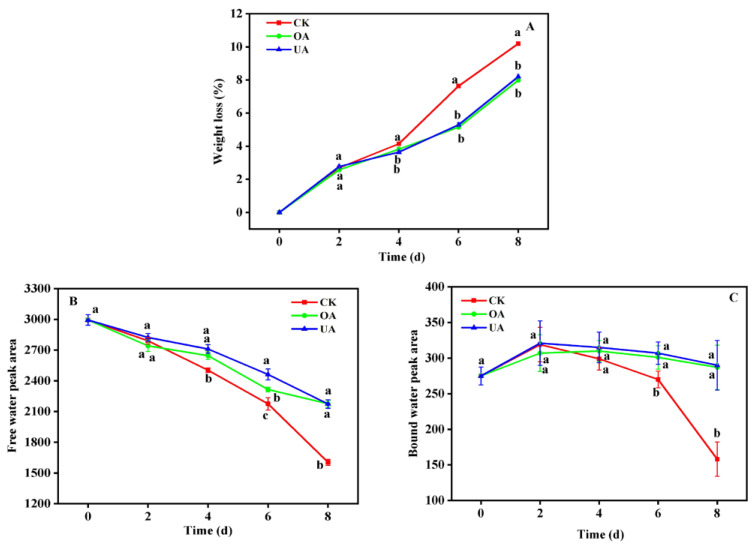
Effects of OA and UA treatments on moisture loss. (**A**) Changes in weight loss during storage. (**B**) and (**C**) were the changes in fruit free water and bound water peak areas under different treatments, respectively, which represent the changes in water molecules status in the fruit. In (**A**–**C**), mean differences between the bars are significant at *p* < 0.05 level for different letters but not significant for the same letters.

In light of the above findings, cuticular wax components may be involved in the water retention process due to their hydrophobic properties and the formation of hydrophobic layers on fruit surfaces. To further verify the role of triterpenoid substances OA and UA in fruit water migration during storage, CK, OA, and UA treatment experiments were conducted, and the water migration process of fruit was measured within eight days by LF-NMR. As can be seen from Figure 5 and Figure 6, based on the inversion of lateral relaxation time (T_2_), it was determined that the content and composition of water inside the fruit were mostly free water (T_23_), and the peak area A_23_ represents the changes in the contents. It was not only that the free water of fruit kept losing over time, but it also went through a process of transforming from free to bound water. Since the CK group had the fastest fruit water loss, there was no significant difference in water content between all groups during the first four days storage due to the transition from free to bound water. As the storage period progressed, the water content of the CK group decreased significantly, and the differences with that of the other two treatment groups (OA and UA) became apparent after four days. In turn, this clearly leads to substantial differences in fruit weight-loss rates. In terms of bound water conversion, OA and UA treatment groups performed significantly better than the CK group, which may be a consequence of a hydrophobic layer that prevents free water from dispersing and encourages its conversion into bound water on the surface of the fruit. However, it is worth noting that there was no significant difference between OA and UA treatment groups in fruit water content change or weight loss change during storage, which indicates that OA and UA blueberry wax components may play similar roles in fruit water migration.

## 4. Discussion

Many of the quality characteristics of fleshy fruit depend on moisture, which makes up a large percentage of their wet weight and plays a crucial role in metabolic activity and quality, such as protein denaturation, enzyme activity, and hardness [28]. After harvesting, the process of absorbing water from the plant roots is terminated, and the consumption of water by various life activities leads to changes in the structure, texture, and the surface of the fruit. In the present study, fruit softening, shrinking, and other phenomena gradually occur as the weight of the fruit decreases during storage, and there is a strong correlation between them [8]. Water loss is a common phenomenon in postharvest fruit, which is easily affected by fruit surface structure. The cuticle is the outermost structure of the fruit, and its hydrophobicity endows the fruit an effective barrier to prevent water loss [29]. Cuticular wax attached to the cuticle surface has been fully confirmed to affect the water permeability of fruit, inhibit non-stomatal water loss, and contribute to the maintenance of storage quality [30,31]. Cuticular wax thickness and total wax content are important factors affecting water loss [32]. Verifying the correlation between cuticular wax and storage-quality wax has been carried out in blueberry fruit [4]. For instance, remove the cuticular wax from the blueberry significantly enhanced fruit water loss and rot rate, decreased fruit sensory quality, and reduced shelf life [4]. Here, as can be observed from Figure 1 and Figure 3, the total wax content decreased continuously, which was negatively related to the weight loss of fruit during storage. Further evidence by analysis of hardness and fruit diameter as well as by scanning electron microscope observation verified that the content of wax in blueberries was closely related to water loss and quality (Figure 3). However, the wax content during cold storage (0.5 °C) has the opposite change trend [20]; the increase in wax content may be a stress reaction against cold stress. Therefore, the variety of storage environments may contribute to different wax deposition in blueberries.

Moreover, considerable effort was also focused on the effect of the wax composition and structure on the permeability of the epidermis [33]. As reported in previous studies, aliphatic VLCFA derivatives could greatly help to enhance the effect of the epidermal transpiration barrier [34,35]. The existence of other wax components such as triterpenoids, which are highly hydrophobic, and can improve the physical stability of water barrier [22]. Likewise, in the current study, although the triterpenoids OA and UA showed first an increase and then a decrease in their content, the correlation analysis further demonstrated that they were closely related to fruit weight loss as well as the amount of cuticular wax (Figure 3). Therefore, the explanation for the correlation between UA content and fruit quality may be linked to their effect on water loss. Moreover, as the substances with the highest contents of waxy triterpenes in blueberry fruit, the droplet contact angles of OA and UA proved their hydrophobic properties. Further evidence by the thin-plate chromatography experiment showed that UA and OA exhibit similar molecular polarity in the solvent systems, with very little polarity. Therefore, triterpenes may play an important role in limiting water loss during storage.

Recently, LF-NMR has been gradually conducted to monitor changes in the moisture status of fruit and vegetables under a variety of conditions due to the advantages of large amplitude range, sensitive phase response, and short detection time [36,37]. The detected T_2_ signal (transverse relaxation time) could display the content and status of water molecules in samples [38]. As mentioned in the results of LF-NMR measurements, water migration and state changes in sweet cherries were highly correlated with water loss and fruit softening [27]. Similarly, in this work, the coating treatments with OA and UA on blueberries demonstrated the potential role of triterpenoids in suppressing water loss. Our study was similar to the previous studies on highbush blueberries, which suggested that the content and proportion of triterpenoids negatively correlated with the water-loss rate [20]. There were no obvious differences between OA and UA treatment in free water and bound water, while they were significantly higher than CK. It can therefore be assumed that the treatment of OA and UA not only suppresses the free water loss but also promotes the process of conversion from free water to bound water. Obviously, this results in lower fruit weight loss. At the end of the storage period, weight loss was markedly lower (by 22%) in the blueberry fruit with UA treatment than in the control fruit. Hence, it is conceivable to suppose that part of the life activities involved in water in fruit may be inhibited, which in turn prolongs the shelf life. This important water migration process that we found was also reported in the blueberries stored at low temperatures [28]. Interestingly, they also pointed out that dehydration was a manifestation of chilling injury.

In addition, there are many factors affecting fruit water loss [39]. Although triterpenoids may have a potential effect on preventing fruit water loss, definitive conclusions cannot be drawn directly. A study by Moggia [6] showed that the samples suffered higher deterioration rates with higher UA content during storage. They found differences in UA and OA content between two different blueberry varieties with different water loss performances, but there was no significant difference in the total amount of triterpenoids. This suggests that triterpenoids may not be the only factor affecting water loss in cuticular wax components. The existence of other cuticular wax components such as alkanes similarly plays an important role in elevating the hydrophobic properties [22,40]. Leide et al. [17] reported that, compared with the wild-type, the cuticular wax of mutant tomato fruit was almost completely devoid of n-alkanes and lost water very easily. Yang et al. [22] reported that water loss in six stored citrus varieties was significantly correlated with cuticle alkanes, and coating with C_28_ alkane could significantly decrease moisture loss. Interestingly, some studies have also shown that in some stored fruit and vegetables, such as tomatoes [17], peppers [13], sweet cherries [18], and peaches [19], the water-loss rate was closely correlated with the ratio of normal alkanes to triterpenes and sterols. However, in this work, the alkanes did not received the necessary attention and research due to their relatively low amount in blueberry cuticular wax. Therefore, factors such as variety differences, cuticle structure and thickness, other cuticular wax components, and even storage environment need to be investigated in further studies.

## 5. Conclusions

In conclusion, cuticular wax is of great significance for the preservation of blueberries after harvesting. Through HPLC content detection and correlation analysis, the content of the triterpenoid substance UA in blueberry cuticular wax was positively correlated with the total wax content, fruit firmness, and fruit diameter. Moreover, UA and OA have small molecular polarities and stable hydrophobic properties, which play important roles in the formation of surface water barriers in fruits. More importantly, the LF-NMR detection of moisture migration in UA- and OA-treated fruit provided substantial evidence that water loss performance was significantly inhibited, and the free and bound water content of the fruit slowly decreased. Our findings will help to reveal the functional role of cuticular wax components in fruit preservation, and they suggest a new perspective in the development of natural packaging to maintain the postharvest quality of blueberries.

## Data Availability

The data used to support the findings of this study can be made available by the corresponding author upon request.
